# High throughput microparticle production using microfabricated nozzle array†

**DOI:** 10.1039/d4ra09032b

**Published:** 2025-03-03

**Authors:** Süleyman Çelik, Ümit Çelik, Ali Koşar, Abdulhalim Kılıç

**Affiliations:** a Department of Molecular Biology and Genetics, Istanbul Technical University 34469 Istanbul Turkey; b School of Civil Aviation, Firat University Elazig 23119 Turkey kilicabd@itu.edu.tr; c Sabancı University Nanotechnology Research and Application Center (SUNUM) 34956 Istanbul Turkey kosara@sabanciuniv.edu; d Faculty of Engineering and Natural Science, Sabancı University 34956 Istanbul Turkey; e Sabancı University, Center of Excellence for Functional Surfaces, and Interfaces for Nano Diagnostics (EFSUN), Faculty of Engineering and Natural Sciences Tuzla Istanbul 34956 Turkey; f Turkish Academy of Sciences Turkey

## Abstract

Polymeric microparticles have triggered critical advancements in drug delivery systems, offering significant improvements in therapeutic efficacy by controlling the delivery while minimizing adverse side effects of the pharmaceuticals. However, conventional microparticle fabrication techniques face several limitations, such as particle size variability, early drug degradation, and production inefficiencies. In this study, we developed a microparticle production system (MPS) in which a precision spraying technology was integrated with a microfabricated nozzle array-based piezoelectric transducer. High-throughput microparticle production was achieved using Poly(d,l-lactide-*co*-glycolide) (PLGA) dissolved in dichloromethane (DCM) and dimethyl carbonate (DMC). The resulting PLGA microparticles exhibited remarkable consistency in size uniformity with an average diameter of 8.9 ± 1.7 μm. Detailed characterization through scanning electron microscopy (SEM) and focused ion beam (FIB) analyses revealed distinct surface and internal structures and demonstrated the effect of solvent volatility on microparticle morphology. Chloramphenicol (CHL) was used as a model drug, and an encapsulation efficiency of 38.7% and a loading efficiency of 16.2% were achieved. The PLGA microparticles showed sustained CHL release and demonstrated effective antibacterial activity against *Escherichia coli* (*E. coli*), highlighting their potential for controlled therapeutic applications. This developed MPS system offers a scalable and efficient approach for producing PLGA-based microparticles with controlled drug release profiles, making it valuable in the industrial-scale production of advanced drug delivery technologies.

## Abstract 2

Microparticle production system utilizing a microfabricated nozzle array and piezoelectric technology enables precise and scalable polymeric microparticle production, offering significant improvements in drug delivery systems.

## Graphical abstract

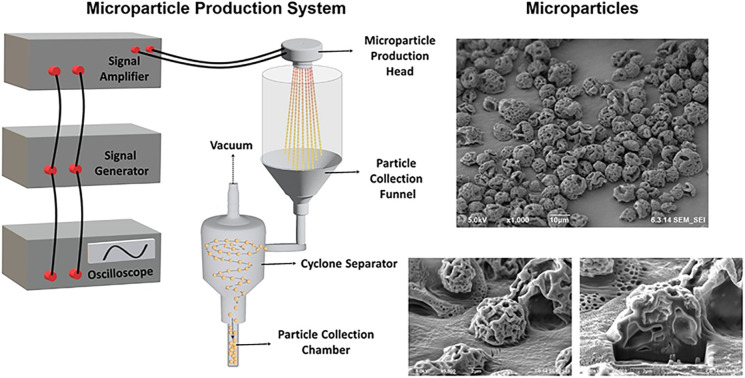

## Introduction

The controlled delivery of pharmaceutical agents remains a critical challenge in modern medicine. Conventional therapeutic approaches typically rely on repeated drug administration to maintain effective concentrations in the body. However, these methods commonly result in fluctuations of drug levels in the bloodstream, potentially leading to periods of suboptimal treatment effects or toxic side effects.^1^ Furthermore, elevated drug concentration throughout the body might cause unintended interactions with healthy tissues and organs, resulting in unpredicted adverse side effects.

Polymeric microparticle-based drug delivery systems offer some advantages over conventional methods by enabling the controlled and sustained release of therapeutic agents, thereby minimizing fluctuations in the plasma concentration, reducing the dosing frequency, and enhancing patient compliance.^2–5^ The encapsulation of drugs within polymer microparticles improves the bioavailability by protecting sensitive compounds from enzymatic or pH-induced degradation and allowing for tailored release profiles that can be adjusted based on the polymer's physical and chemical properties, such as molecular weight and composition.^6,7^ Additionally, surface modifications facilitate targeted drug delivery to specific tissues or cells, thereby reducing systemic toxicity and enhancing therapeutic efficacy.^8,9^ The versatility of microparticles enables the encapsulation of a wide range of therapeutic agents, including hydrophilic and lipophilic drugs, proteins, and nucleic acids, broadening their applicability across various medical fields^10,11^ Furthermore, the controlled release mechanisms inherent to polymer microparticles improve patient compliance by reducing the dosing frequency and minimizing systemic toxicity by targeting drug delivery to specific sites.^5,12^ Polymers such as PLGA, allow for safe *in vivo* applications without eliciting adverse immune responses and provide controlled degradation into non-toxic lactic and glycolic acid derivatives, ensuring safe drug release.^13,14^ Polymeric materials also enable innovative manufacturing techniques that enhance the stability and uniformity of drug delivery systems, making them a preferred choice in recent pharmaceutical applications.^15^ Overall, the integration of polymer microparticles into drug delivery strategies leads to a significant advancement in achieving effective therapeutic outcomes while addressing the limitations associated with conventional dosage forms.

The growing demand for advanced drug delivery systems capable of providing controlled and sustained release of therapeutic agents has demonstrated their remarkable potential.^6,14,16,17^ These technologies enhance drug bioavailability by regulating the amount and duration of drug release while enhancing treatment efficacy and minimizing side effects while reducing toxicity.^18,19^ Controlled drug release systems are typically engineered by embedding drugs within polymer-based biocompatible and biodegradable matrices.^13,20^ The formulation of these matrices in microparticle morphology offers the potential for safer and more effective therapeutic outcomes. These micro-scale designs provide multiple advantages, including protection against early drug degradation, prolonged circulation in the bloodstream, and enhanced tissue-specific delivery.^18^ Recent advances in microparticle engineering accelerate the research efforts of interdisciplinary research in pharmaceutical sciences, polymer chemistry, nanotechnology, cellular biology, and medicine in optimizing the composition, size, surface properties, and architecture of microparticles for enhanced therapeutic outcomes.^21,22^ polylactic acid (PLA) and poly(d,l-lactide-*co*-glycolide) (PLGA) are preferred polymers for controlled drug delivery systems due to their biocompatibility and biodegradability.^23^ Various methods for producing polymeric microparticles have been developed, including emulsion-solvent evaporation,^2^ double emulsion (W/O/W),^3^ spray drying, ionic gelation,^24^ coacervation,^24^ and microfluidic devices.^25^ While these traditional microparticle fabrication methods offer distinct advantages, they also introduce various limitations. The solvent extraction/evaporation method is known as suitable for hydrophobic drugs but often fails to achieve the desired precision in particle size homogeneity, drug loading efficiency, and release kinetics.^2,26,27^

The coacervation technique, advantageous for water-soluble drugs and biomolecules, faces challenges related to high organic solvent requirements and frequent agglomeration.^2,28,29^ The spray-drying technique, known for rapid processing and scalability, presents challenges, including drug degradation due to high temperatures and product loss through particle adhesion and agglomeration.^2,30–32^ In addition, spray drying, while efficient for large-scale production, involves high processing temperatures that can degrade heat-sensitive compounds, making it less suitable for thermolabile drugs.^26,33^ Emulsion-solvent evaporation is widely used due to its simplicity and ability to encapsulate a broad range of drugs; however, it often results in polydisperse particles and limited control over morphology.^34,35^ Microfluidic devices offer precise control over the particle size and composition, but their low throughput limits their applicability in real-life applications.^36,37^ Similarly, electrospray enables monodisperse particle production but requires high voltages and complex operational setups, posing challenges for scalability.^38,39^ In this study, an advanced atomized spray-drying microparticle production system (MPS) enabling high-throughput production of uniformly sized microparticles was developed for controlled drug delivery systems. In this system, a precisely microfabricated uniform spray nozzle array on a silicon wafer surface fabricated through microfabrication techniques was integrated with a piezoelectric transducer to generate consistent microdroplets upon excitation. A custom-made 3D-printed cyclone separator was attached to the system for particle drying and collection. PLGA dissolved in dichloromethane (DCM) and dimethyl carbonate (DMC) was used to produce microparticles using MPS. Chloramphenicol (CHL) was used as a model drug to produce drug-loaded microparticles. The internal structures of produced PLGA microparticles were characterized by focused ion beam (FIB) and scanning electron microscopy (SEM). The performance of drug-loaded PLGA microparticles was determined by assessing the release kinetics through UV-vis spectroscopy and analyzing their antibacterial activity. Our proposed system was developed to overcome these limitations by enabling high-throughput production of uniformly sized microparticles with minimal manual intervention. This method ensures precise control over the particle size and morphology while operating at room temperature, thereby reducing the risk of drug degradation, which is a significant drawback in spray drying. Furthermore, the ability to fine-tune solvent evaporation dynamics enhances polymer solidification control, improving particle stability and drug release kinetics. The scalability of this system makes it suitable for both laboratory research and industrial pharmaceutical applications, providing a versatile platform for microparticle-based drug delivery and biomedical applications.

## Materials and methods

### Materials

Poly(d,l-lactide-*co*-glycolide) (PLGA, (C_4_H_4_O_4_)_*m*_(C_6_H_8_O_4_)_*n*_), chloramphenicol (CHL, C_11_H_12_Cl_2_N_2_O_5_) and the solvents dichloromethane (DCM, CH_2_Cl_2_), dimethyl carbonate (DMC, C_3_H_6_O_3_) were purchased from Sigma-Aldrich (St. Louis, MO, USA) and used without further purification. The ultrasonic transducer (HS-R8351505, Guangdong, CN) was sourced from He Shuai Company. Conductive epoxy adhesive (EPO-TEK® H20E, MA, USA) was purchased from Epoxy Technology, Inc., and insulating epoxy (Araldite AV 138 M-1 HV/Hardener HV998-1, Basel, CH) was acquired from Huntsman Advanced Materials.

### Preparation of plain and drug loaded solutions

The performance of the advanced system was evaluated using PLGA with an 85 : 15 lactide ratio (catalog number 430471, Sigma-Aldrich) for microparticle production. Two distinct polymer solutions were formulated to prepare PLGA microparticle solutions using the organic solvents DCM and DMC, commonly employed to dissolve PLGA. Briefly, 700 mg of PLGA was weighed and transferred into two clean, dry 250 mL glass beakers. Subsequently, 100 mL of DCM (catalog number 34856, Sigma-Aldrich) was added to one beaker, and 100 mL of DMC (catalog number 517127, Sigma-Aldrich) was added to the other using graduated cylinders. Each mixture was continuously stirred with a magnetic stirrer at room temperature overnight until PLGA was completely dissolved in both solvents. The resulting 7 mg per mL PLGA solutions in DCM and DMC were stored separately in tightly sealed glass containers to prevent solvent evaporation until further use. Drug loading studies were performed exclusively with PLGA solutions prepared in DCM. For the production of drug-loaded PLGA microparticles, chloramphenicol (CHL) (catalog number C0378, Sigma-Aldrich) was incorporated into the previously described PLGA solution in DCM. In this process, 35 mg of CHL was weighed, dissolved in 1 mL ethanol (catalog number 1.00974, Sigma-Aldrich), and mixed into a 9 mL PLGA solution in DCM, achieving a final concentration of 3.5 mg of CHL per mL. Continuous stirring was maintained to ensure uniform drug distribution within the polymer.

To determine the encapsulation efficiency (EE) and loading efficiency (LE), 750 μg of drug-loaded PLGA microparticles were dissolved in 1 mL DCM. The drug concentration in the resulting solution was measured using a validated spectrophotometric method at 278 nm wavelength (Thermo Scientific NanoDrop 2000c, USA).^40^ The encapsulation efficiency (EE%) and loading efficiency (LE%) were calculated using the following equations:^41^
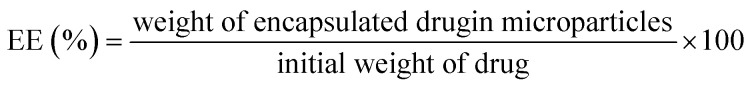

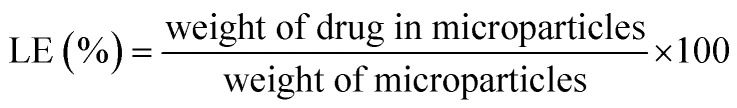


*In vitro*, drug release studies were performed by washing the drug-loaded microparticles three times with 1× PBS buffer before use. The washed microparticles were then suspended in 1× PBS at 1 mg mL^−1^ concentration and transferred into Eppendorf tubes. The samples were incubated at 37 °C in a shaking incubator to maintain optimal dissolution conditions. At predetermined time intervals, aliquots were collected and analyzed using UV-vis spectrophotometry at a wavelength of 278 nm. The experiments continued until the drug was completely released from the microparticles.

### Design and assembly of microparticle production head

**Fig. 1:**
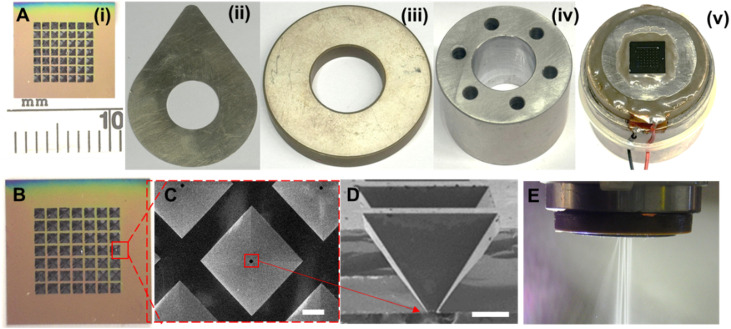
Assembly of the microparticle production head components; (A) images of microparticle producer head components: (i) microfabricated spray nozzle array, (ii) metal disc, (iii) ultrasonic (piezoelectric) transducer, (iv) solution reservoir, (v) assembled microparticle production head. (B) A magnified image of a microfabricated spray nozzle array. (C and D) SEM image of a single nozzle (scale bars show 200 μm). (E) Image of an operating microparticle production system.

The microparticle production head consists of multiple components assembled layer by layer, as demonstrated in Fig. 1. Briefly, the fabrication process began with a microfabricated uniform spray nozzle array (Fig. 1A-i) created through silicon wet etching. Initially, a 550 μm thick double-sided polished (100) silicon wafer was coated with a 200 nm layer of silicon nitride (Si_3_N_4_) on both sides using plasma-enhanced chemical vapour deposition (PECVD) (Oxford PlasmaLab System 100) as a mask. Predefined micro-porous nozzle patterns were transferred onto the wafer surface through optical lithography (Midas/MDA-60MS Mask Aligner). Subsequently, the silicon nitride mask was etched using deep reactive ion etching (DRIE) (Oxford PlasmaLab System 100 ICP 300). After silicon nitride patterning, a piranha solution was used to remove the remaining photoresist. The wafer subsequently underwent anisotropic wet etching in a 40% (W/V) potassium hydroxide (KOH) solution at 80 °C, with the backside silicon nitride layer serving as a protective mask.

Following the completion of silicon etching, a buffered oxide etch (BOE) was used to remove the remaining silicon nitride. This process was optimized to produce uniform microparticles with consistent size and distribution. A 7 × 7 micro-nozzle array, shown in Fig. 1B, was fabricated on a silicon wafer with hole diameters of 22 ± 0.5 μm (Fig. 1C and D). The microfabricated spray nozzle array was attached to a metal disc with a 10 mm central hole using a piezo adhesive (Araldite AV 138 M-1 HV/Hardener HV998-1) (Fig. 1A-ii) to stabilize the nozzle array and ensure a leak-free operation. An ultrasonic (piezoelectric) transducer (PZT-8) (Fig. 1A-iii), equipped with contact electrodes, was affixed to the metal body using epoxy (EPO-TEK® H20E). The piezo disk moves in the thickness expansion mode to vibrate the micro-nozzle array vertically to produce microdroplets. A stainless steel 316 reservoir (Fig. 1A-iv) was attached to the piezoelectric disk to supply the necessary organic solvent–polymer solution. The assembled microparticle production head can be seen in (Fig. 1A-v).

### Design and components of microparticle production system

**Fig. 2:**
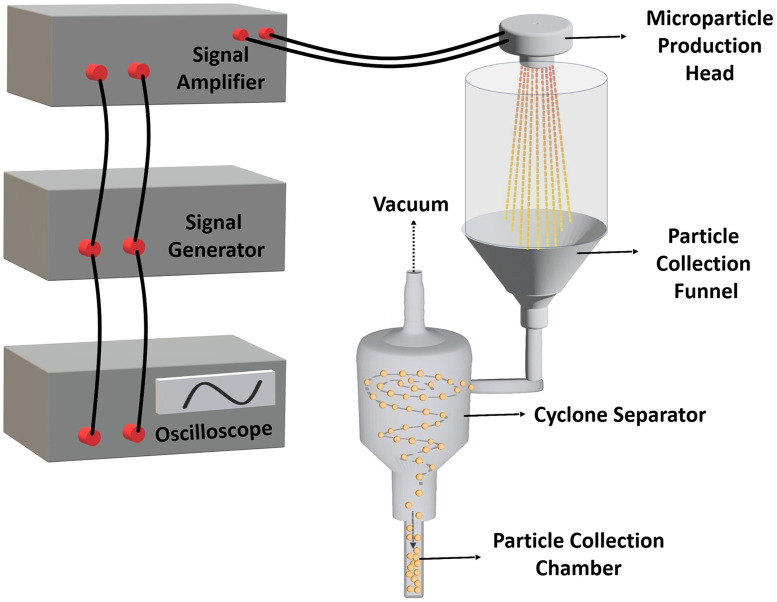
Design and components of the micro-jet nozzle-based microparticle production system.

The microparticle production system, illustrated in Fig. 2, employs a micro-jet nozzle array combined with piezoelectric transducer disks and a microfabricated spray nozzle array to transform a polymeric solution into fine microdroplets. The system consists of two main units: the electronic control unit and the particle production-collection unit. The electronic unit includes a signal generator (Agilent 33220A) to create the required waveform driven to the piezoelectric disk, a high-voltage amplifier (Nanomagnetics Instruments) to amplify the signal generator output, and an oscilloscope (Tektronix TDS3034B) to monitor the driven signals. The particle production collection unit comprises the microparticle production head and a 3D-printed cyclone separator. The process began when electrical stimulation triggered the high-frequency vibrations in the microparticle production head. The piezoelectric disk moved vertically back and forth, accelerating the polymeric solution toward the jet nozzles. At resonance frequencies, these waves fragmented the liquid into microdroplets through atomization. The microfabricated spray nozzle array regulated the liquid ejection to ensure uniform droplet size. This piezoelectric transducer-based system operated at a resonance frequency of 46.7 kHz and an amplitude of 65 V, producing microparticles with similar sizes and morphologies through controlled vibrations. The system was driven at its resonance frequency to achieve the maximum atomization efficiency.^42^ The produced microdroplets were collected by a 3D-printed funnel, which directed them to the cyclone separator. An airflow expelled *via* a vacuum system allowed microparticle formation through rapid droplet solidification during the time of flight. Finally, the microparticles were kept in a collection chamber at the end of the process.

### Characterization of PLGA microparticles

The surface and internal structure of PLGA microparticles were systematically investigated using scanning electron microscopy (SEM) and focused ion beam (FIB) techniques (JEOL JIB-4601 MultiBeam FIB-SEM). Prior to imaging, the microparticles were sputter-coated with a thin layer of Au–Pd using a Ted Pella 108 Auto Sputter Coater to enhance the conductivity and prevent charging under the electron beam. Energy-dispersive X-ray spectroscopy (EDS) integrated with SEM (Oxford X_Max) was employed to determine the elemental composition and distribution within the particles, confirming the chemical integrity and homogeneity.

Fourier-transform infrared spectroscopy (FTIR), performed using the Shimadzu IRAffinity-1S, was conducted to identify the chemical bonds and interactions within the microparticles. To characterize the particle size distribution, SEM images were captured from randomly selected regions of the microparticles produced using both DCM and DMC as solvents. These images were subsequently analyzed using the ImageJ software, and histograms were generated to visualize the particle size distribution data, which were obtained by generating histograms from the SEM images for both solvent types.

### Methodology and setup for investigating the effects of microparticles on bacteria

Bacterial cultures were established to investigate the effects of plain and drug-loaded microparticles on bacterial growth. The drug release performance of the antibiotic-containing microparticles was evaluated in a liquid Luria-Bertani (LB) medium containing *Escherichia coli* (*E. coli* K12) bacteria. Initially, *E. coli* samples were inoculated onto solid agar media at 1.5% (w/v) concentration and incubated at 37 °C for 24 hours to obtain single colonies. Individual colonies from each plate were then transferred into 3 mL of liquid LB medium and incubated overnight at 37 °C in a shaking incubator to ensure optimal bacterial growth. Following overnight incubation, each culture was divided into four groups with three replicates to evaluate different treatment conditions. The first group served as the negative control (*E. coli*) with no additional substances (*i.e.*, microparticles, antibiotics), allowing measurement of baseline bacterial growth. The second group (plain PLGA + *E.coli*) received a solution containing 7 mg per mL plain PLGA microparticles to examine the impact of plain PLGA microparticles and their degradation products on bacterial growth. The third group (drug-loaded PLGA + *E. coli*) was treated with 7 mg per mL PLGA microparticles loaded with 16.2% CHL. This group was designed to study the combined effects of PLGA microparticles and released CHL on bacterial growth, as PLGA degradation could facilitate sustained antibiotic release, potentially enhancing antimicrobial efficacy. The fourth group (drug only) received 1.14 mg per mL CHL to assess the direct impact of the antibiotic, serving as a positive control to compare the effectiveness of CHL-loaded microparticles with free CHL. Prior to use, the drug-loaded microparticles were washed three times with 1× PBS buffer to remove any non-encapsulated drugs from the surface, ensuring that only the encapsulated drug would be released into the medium.

### Statistical analysis

Data are presented as mean ± SD. A significant difference was considered at the level of *p* < 0.05 (*n* ≥ 3) and indicated with asterisks (*). A two-way analysis of variance (ANOVA) was employed to evaluate differences among the experimental groups after assessing normality with Tukey's test. Significance was set at *p* < 0.01 (**), *p* < 0.001 (***), and *p* < 0.0001 (****).

## Results

In this study, a microparticle production system (MPS) was developed by integrating precision spraying technology with a microfabricated nozzle array-based piezoelectric transducer. Upon completion of the assembly, the microparticle production head demonstrated efficient operation, as shown in Fig. 1E. The 7 × 7 nozzle array (as shown in Fig. 1B) expelled a fine mist of solution, resulting in the production of microparticles.

**Fig. 3:**
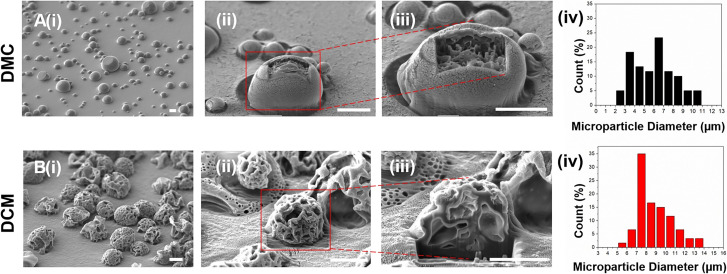
Comparison of the effects of solvents (A) DMC and (B) DCM used in the production of PLGA microparticles on the surface and internal morphology of the microparticles: SEM image showing the (i) general distribution of microparticles, (ii) surface morphology, (iii) internal morphology of microparticle cross-section obtained by FIB and (iv) size distribution histograms of microparticles (scale bars show 5 μm).

The performance of the proposed system was evaluated by producing PLGA-based microparticles, as PLGA has been widely used in drug delivery systems.^3^ DCM and DMC were used to evaluate the effects of solvents on the surface and internal morphology. These solvents differ significantly in their evaporation rates, which play a crucial role in microparticle production. DCM, with its lower boiling point (39.6 °C) and higher volatility, evaporates quickly, facilitating faster solvent removal and promoting the formation of more uniform microparticles. In contrast, DMC, which evaporates more slowly due to its higher boiling point (90 °C) and lower volatility, allows more time for particle interaction and coalescence, often resulting in larger and more variable particle sizes. These distinct evaporation properties substantially influence both the production process and the characteristics of the final microparticles. Microparticles were produced using DCM and DMC as solvents and were characterized by focused ion beam scanning electron microscopy (FIB-SEM) to reveal their surface and internal morphology (Fig. 3). SEM provided detailed images of the surface morphology, revealing the shape, texture, and size of the microparticles. FIB was used to create precise cross-sections, enabling examination of internal morphology, including the core–shell architecture and porosity. These analyses were crucial for assessing the uniformity, encapsulation efficiency, and controlled release properties of the microparticles. The SEM images in Fig. 3 show a clear difference in the surface morphology depending on the solvent used. Microparticles produced with DMC (Fig. 3A-i) have smooth, non-porous surfaces due to slower evaporation of the solvent, while the microparticles produced with DCM show a porous surface behaviour (Fig. 3B-i).

The rapid evaporation of DCM is the reason for forming porous structures with interconnected channels and cavities on their surfaces. Given the porous architecture, these microparticles are particularly suited for diffusion-controlled drug release systems, as the high surface area facilitates drug diffusion from both the surface and the inner matrix of the particles.

FIB cross-sectioning (Fig. 3A-ii and iii) was employed to further reveal the internal structure of microparticles. It can be seen that the internal structures of the microparticles have porous and lamellar internal structures, regardless of the solvent. However, examination of the internal structure through FIB cross-sectioning reveals that the lamellae in microparticles prepared with DMC were thinner and less robust than those in microparticles prepared with DCM, as the DMC-produced microparticles rapidly collapsed during the FIB cutting process. This suggests that while DCM facilitates the formation of denser and more mechanically stable lamellar structures, DMC leads to the production of finer and less stable lamellar formations, likely due to the solvent's influence on polymer chain entanglement and crystallization during the evaporation process. These findings highlight the significant impact of solvent choice on both surface and internal morphologies of PLGA microparticles, with potential implications for drug delivery systems, where the surface and internal architecture critically affect the drug release kinetics and stability.

Fig. 3A-iv and B-iv show the size distribution of PLGA microparticles produced using DCM and DMC. Particles produced with DCM have an average size of 8.9 μm with a standard deviation of 1.7 μm, indicating relatively uniform particle sizes, mostly between 6 and 10 μm in diameter. In contrast, DMC-produced particles have an average size of 6.3 μm but a higher standard deviation of 2.9 μm, indicating a wider size range from 2 μm to 11 μm, with a peak around 6–8 μm. While DMC produces smaller particles on average, it fails to achieve the same size homogeneity as DCM. It is important to note that the particles are initially ejected homogeneously from the nozzle head, but some tend to re-coalesce during flight time after spraying. This effect is more pronounced in DMC due to its higher boiling point and slower evaporation rate, allowing particles more time to interact and merge, resulting in a broader size distribution.

**Fig. 4:**
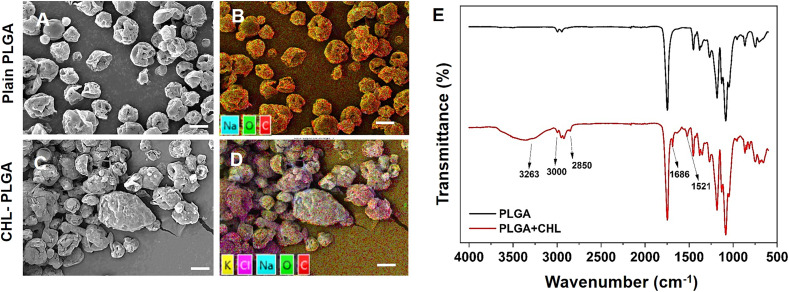
Characterization of drug loading within PLGA microparticles. SEM images of (A) plain and (C) CHL-loaded PLGA microparticles. EDS spectra of (B) plain and (D) CHL-loaded microparticles displaying chlorine content. (E) Comparison of FTIR spectra of PLGA and CHL-loaded PLGA microparticles (scale bars show 10 μm).

DCM was selected as the solvent for producing drug-loaded porous PLGA microparticles. Chloramphenicol (CHL), a broad-spectrum antibiotic used in treating bacterial infections, was chosen as the model drug to be encapsulated within the PLGA microparticles. The SEM images in Fig. 4. show the morphological characteristics of plain PLGA microparticles (Fig. 4A) and CHL-loaded PLGA microparticles (Fig. 4B). Both sets of microparticles exhibit a relatively uniform and porous surface texture with spherical shapes and minor agglomerations. No significant morphological changes are observed between the plain and drug-loaded microparticles, indicating that the drug-loading process does not significantly alter the physical characteristics of the PLGA microparticles. This consistency in the morphology suggests that the integrity and surface properties of the microparticles are maintained after drug loading, which is crucial for ensuring reliable performance in drug delivery applications.

To verify drug loading, elemental analysis was performed using EDS. The CHL contains chlorine (Cl) in its chemical structure, whereas it is absent in the plain PLGA structure. The EDS mapping results are in Fig. 4D shows the presence of the Cl element in the CHL-loaded microparticles, which is absent in the plain PLGA microparticles (Fig. 4B). Cl indicates the drug incorporated within the PLGA matrix, confirming successful drug loading.

Fig. 4E presents the FTIR spectra, comparing plain PLGA microparticles (black line) with CHL-loaded microparticles (red line). The spectra display several distinct peaks corresponding to specific functional groups, offering insights into the chemical interactions and potential modifications induced by CHL loading. A broad peak observed around 3263 cm^−1^ in the CHL-loaded PLGA marks the presence of hydroxyl group (–OH), significantly reduced in the plain PLGA. This suggests an interaction between CHL and the –OH groups during treatment. Peaks at 3000 cm^−1^ and 2850 cm^−1^, present in both spectra, are attributed to C–H stretching vibrations.^40^ The relative intensity of these peaks is higher in the CHL-loaded PLGA, indicating an increase in the aliphatic hydrogen content, possibly due to solvent interaction or residue. The prominent peak at 1686 cm^−1^ in CHL-loaded spectra corresponds to the carbonyl (C=O) stretching of ester groups in CHL. The intensity of this peak is notably higher in the CHL-loaded PLGA, suggesting an alteration in the carbonyl (C=O) environment, potentially due to solvent-induced changes in polymer conformation. The peak at 1521 cm^−1^, more pronounced in the CHL-loaded PLGA, is associated with bending vibrations of C–H bonds not present in the plain PLGA. This suggests potential structural modifications or interactions facilitated by CHL. In the region below 1500 cm^−1^, several peaks correspond to various bending and stretching vibrations of the PLGA backbone. The analysis indicates no significant chemical changes in the PLGA structure due to CHL loading. The primary polymer backbone and functional groups remain primarily intact, confirming that CHL acts more as a physical agent rather than inducing chemical modifications. This analysis confirms their chemical composition and detects any potential modifications during the manufacturing process. These combined analytical techniques are instrumental in providing a robust understanding of both the physical and chemical properties of the PLGA microparticles, supporting their suitability for advanced drug delivery applications.

**Fig. 5:**
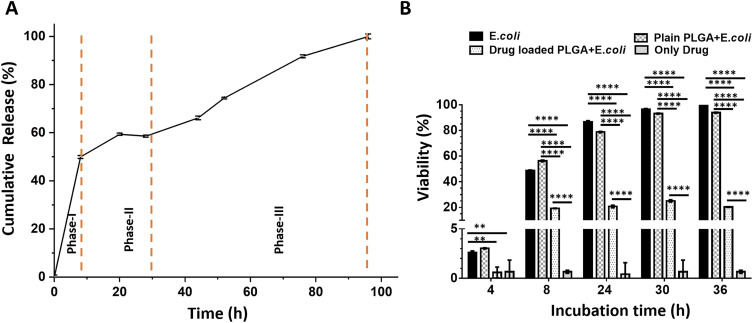
*In vitro* drug release performance and antibacterial activity of drug-loaded PLGA microparticles. CHL was used as a model drug loaded in PLGA microparticles. (A) The absorbance at 278 nm was measured to characterize the CHL release performance of the microparticles at different time points. Phase I showed the burst release, while phase II was used to show the sustained release condition. Phase III showed the rapid release upon degradation of PLGA microparticles. (B) *E. coli* were exposed to drug-loaded and plain PLGA microparticles, and OD 600 values were measured at different time points to calculate the viability. *E. coli* growth was used as a negative control, and the only drug was used as a positive control. Data was normalized to the negative control, set at 100%. A two-way ANOVA analysis was used to calculate the significant change between the groups (*p* < 0.01 (**) and *p* < 0.0001 (****)).

Fig. 5A shows the *in vitro* drug release profile from drug-loaded PLGA microparticles for 100 hours. The release reaches 100% by the end of this period, indicating complete drug release. The drug release profile demonstrates a characteristic triphasic release pattern. A triphasic release profile comprises three distinct phases: an initial burst release phase (Phase I), followed by a slower, sustained release phase (Phase II), and concluding with a final rapid release phase (Phase III).^43,44^

During the first phase (Phase I), a rapid release is observed, characterized by an initial “burst release.” This phenomenon is likely attributed to the dissolution of drug molecules located near the surface of the particles. The second phase (Phase II), occurring between approximately 10 and 30 hours, exhibits a slower, sustained release rate, which corresponds to the controlled diffusion of the drug within the particle matrix. The third phase (Phase III), beginning around 30 hours and continuing to 100 hours, reveals an accelerated release, presumably resulting from the hydrolytic degradation of the PLGA matrix and the subsequent release of the remaining encapsulated drug.

Fig. 5B shows the viability of *E. coli* under different treatment conditions. The viability of *E. coli* in the untreated and plain PLGA groups reaches 100% by 36 hours, indicating that plain PLGA microparticles do not affect bacterial growth. In contrast, drug-loaded PLGA microparticles significantly reduce the viability, demonstrating effective antibacterial activity.

The experiment was conducted using OD_600_ measurements from samples collected at 4, 8, 24, 30, and 36 hours. In the “*E. coli*,” the viability begins at a very low level at 4 hours and progressively increases over time, reaching nearly 100% by 30 hours and maintaining this level up to 36 hours, which reflects the typical growth curve of *E. coli*. In the “Only Drug” group, the OD_600_ value remains negligible, indicating no bacterial growth and the direct inhibitory effect of the antibiotic. The “Plain PLGA + *E. coli*” group shows an increase in *E. coli* cell density over time, similar to the control group, and reaches approximately 100% viability at 30 hours. This suggests that PLGA microparticles are biocompatible and do not interfere with bacterial growth. In the “Drug-loaded PLGA + *E. coli*” group, viability remains low throughout the study. During the first 8 hours, the insufficient concentration of CHL allows *E. coli* to grow slowly. However, once the drug reaches an effective concentration, bacterial growth is effectively inhibited. This experiment demonstrates that CHL-loaded PLGA microparticles successfully release the drug over the specified period, providing sustained antimicrobial activity by preventing a significant increase in bacterial viability. Statistical analyses were performed using two-way ANOVA to evaluate differences among the experimental groups, and the results are presented in Fig. 5B for detailed interpretation.

## Discussion

The findings of this study present the effectiveness of a developed microparticle production system (MPS) for producing PLGA-based microparticles optimized for controlled drug release applications. The MPS system utilizes a piezoelectric transducer integrated with a microfabricated nozzle array, enabling the consistent production of microparticles. The microparticle size is critical since it directly affects the drug release kinetics, bioavailability, and overall therapeutic efficacy. Smaller microparticles generally exhibit faster drug release due to their higher surface area-to-volume ratio. In contrast, larger particles may enable a more sustained release profile. The regulation of the particle size in MPS developed in this study can be explained by multiple factors. The first one can be attributed to the synergistic effect of the pore size of microfabricated nozzle arrays and the frequency of piezoelectric transducers, which initiate the microparticle formation through spraying. In the MPS in this study, the diameter of the microfabricated nozzle (22 μm) directly influences the droplet size and, thus, the final microparticle dimensions, which is approximately 9 μm. The formation of the smaller diameter microparticles compared to the nozzle array size demonstrates the effect of the piezoelectric excitation frequency (46.7 kHz), which plays a crucial role in controlling the droplet breakup, thereby affecting the particle size homogeneity. The second factor can be explained by the evaporation rate of the solvent type used for the dispersion of the polymer. In this study, the evaporation rate of the solvent is assessed by the produced microparticles dichloromethane (DCM) and dimethyl carbonate (DMC).^42^ The faster evaporating solvents (DCM) produce smaller sizes and more porous microparticles, while slower evaporating solvents (DMC) lead to denser and larger microparticles.^45^ These parameters can provide a comprehensive approach for controlling the particle size using MPS, which is essential for optimizing drug delivery systems. It is also important to note that the contribution of the viscosity and concentration of the polymer solution needs to be considered since more significant polymer concentrations increase the viscosity, resulting in larger microparticles.

The solvent selection is critical in determining the particle morphology. We selected DCM and DMC in this study due to the fact that PLGA is soluble in these solvents as well as due to their distinct evaporation rates and polarities. In fact, we observed that the selected solvent plays a crucial role in determining the particle size and morphology. DCM, characterized by its low boiling point and high volatility, facilitates rapid solvent evaporation, forming smaller, highly porous microparticles.^43^ Conversely, with its higher boiling point and slower evaporation rate, DMC allows for prolonged polymer reorganization, resulting in larger, denser, and less uniform particles. The mechanism can be further explained by the phase separation kinetics, where fast-evaporating solvents such as DCM promote rapid polymer precipitation, resulting in porous microparticles with interconnected channels. On the other hand, slow-evaporating solvents, DMC, lead to the gradual solidification of polymer chains, forming smooth and dense structures with reduced porosity. A solvent–polymer interaction model incorporating the diffusion-limited evaporation and phase separation dynamics could be developed to further elucidate the relationship between the solvent properties and particle morphology. For instance, a Fickian diffusion model could quantify solvent evaporation rates, while a phase separation framework, such as Flory–Huggins theory, could explain polymer precipitation dynamics. In future studies, a systematic variation of solvent composition and evaporation rates would certainly provide deeper insights into the microparticle formation mechanisms and enable the rational design of tailored drug delivery systems. The mutual effects of these two solvents in a mixture can be advantageous in having optimized microparticles, which suggests a balance between rapid and sustained drug release, where the DCM-induced porosity enhances drug diffusion while DMC promotes particle stability. Additionally, both solvents efficiently dissolve PLGA, ensuring homogeneous polymer dispersion during microparticle formation, while their biocompatibility and minimal toxicity post-processing make them suitable for pharmaceutical applications. This approach represents a promising avenue for future studies in optimizing microparticle-based drug delivery platforms.

This system was inherently designed to be scalable. The production capacity of the proposed system could be further enhanced by varying the system parameters. We collected approximately 150 mg microparticles per hour using the current setup. However, significant improvements in production efficiency could be achieved through several modifications, such as increasing nozzle array density. Additionally, operating multiple units in parallel and optimizing flow rates alongside piezoelectric transducer frequencies could further boost productivity and scalability. With these optimizations, industrial-scale production, such as several hundred grams per day, could be achieved, making this system a viable candidate for large-scale pharmaceutical and biomedical applications.

These easily fine-tuned atomization process and various polymer compositions make this system advantageous for generating microparticles capable of encapsulating active pharmaceutical ingredients. In addition, these microparticles can be designed to provide controlled drug release over a desired period, thereby improving patient compliance and therapeutic outcomes.

Compared to traditional microparticle production methods such as solvent evaporation, spray drying, and microfluidic chips, the MPS system offers several distinct advantages. Solvent evaporation techniques are labor-intensive and often result in a broad range of particle size.^3^ Spray drying, while effective in producing fine particles, typically requires high temperatures that can potentially degrade sensitive pharmaceutical compounds.^2^ The microfluidic chip approach, renowned for its precision, is constrained by low throughput and complexity, making it less practical for large-scale production.^44^

Overall, this study proves that the MPS system represents an effective platform for producing PLGA microparticles and offers a promising approach to drug delivery applications. Potential improvements include increasing encapsulation efficiency and refining release control mechanisms. Future research could focus on optimizing formulation parameters and evaluating the system's adaptability across various therapeutic applications such as targeted drug delivery, cancer treatment, and localized disease treatment. The unique properties of these microparticles, including their precise size and composition, may provide significant advantages in enhancing both therapeutic efficacy and safety.

## Conclusions

The MPS system developed in this study offers a promising platform for producing PLGA-based microparticles for controlled drug release. The system demonstrates capabilities by producing uniform microparticles through high-throughput processes with minimal manual intervention. Its key advantages include scalable production and the ability to achieve consistent results suitable for clinical applications. The system enables efficient mass production by significantly reducing production times and associated costs. A critical feature of the system lies in its controlled solvent evaporation technique, which effectively minimizes solvent residues and consequently enhances the safety and efficacy of the produced microparticles. These characteristics directly address current demands within the pharmaceutical industry for reliable, consistent, and scalable production methodologies. By overcoming limitations inherent in traditional techniques, the MPS system offers an alternative for advanced drug delivery applications.

In conclusion, the MPS consistently produces microparticles with a mean diameter of 8.9 ± 1.7 μm, achieving an encapsulation efficiency of 38.7% and a loading efficiency of 16.2% for CHL. The system's notable attributes include rapid drying capabilities and scalability, making it appropriate for both laboratory and industrial production conditions. Ongoing optimization efforts are anticipated to further improve its performance in PLGA microparticle production, with future research efforts focusing on expanding applications in controlled drug release and biomedical therapeutics.

## Data availability

The data supporting this study have been included in this article.

## Author contributions

Süleyman Çelik: conceptualization, investigation, methodology, writing of original draft. Ümit Çelik: investigation & writing. Ali Koşar: supervision, project administration, reviewing & editing. Abdulhalim Kılıç: supervision, reviewing & editing. All authors have approved the final version of the manuscript.

## Conflicts of interest

There are no conflicts to declare.
